# Opportunities and challenges for Registered Reports in ecology and evolution

**DOI:** 10.1038/s41467-022-32900-1

**Published:** 2022-11-28

## Abstract

*Nature Communications* is now welcoming Registered Report submissions from all fields of research (read our editorial here), and we want to encourage submissions from the ecology and evolutionary biology fields. To introduce this format to researchers in those fields, we interviewed two founding members of the Society for Open, Reliable, and Transparent Ecology and Evolutionary Biology (SORTEE), a network of researchers aimed at improving research practices in ecology, evolutionary biology, and related fields: *Shinichi Nakagawa* (Professor of Evolutionary Ecology and Synthesis at the University of New South Wales, UNSW) and *Rose O’Dea* (Secretary of SORTEE, postdoctoral researcher and fellow at the Wissenschaftskolleg zu Berlin). Below, they share their thoughts on how the fields of ecology and evolutionary biology can advance in reproducibility and transparency.

Can you tell us about your involvement in open science and transparency initiatives?

**SN:** I first clearly recognised poor and opaque reporting of methods and results in scientific papers when I conducted my first meta-analysis during my PhD (2003–2007). At that time, I thought there was nothing much I could do to change scientific reporting for the better. But it all changed when Tim Parker, who is also a behavioural ecologist and meta-analyst (Whitman College, USA), visited me at the University of Otago, Dunedin, New Zealand, in 2014. When we took a stroll at my field site, Dunedin Botanic Garden, Tim asked me to join him in writing a piece highlighting how ecologists and evolutionary biologists can learn to improve our science from psychology and medicine, where open science and transparency initiatives were just taking off^1^.

Then, this article led us and Jessica Gurevitch (Stony Brook, USA) to co-organise a workshop at the Center for Open Science (COS) in Charlottesville, USA in 2015 (when I moved to UNSW Sydney, Australia, and very fortunately, Rose joined my lab). This workshop hosted the Editors-in-Chief or representatives from 30 journals in the field of ecology and evolution, producing seven editorials advocating for improvements in the quality of reporting. There I luckily met Fiona Fidler (Melbourne, Australia). In 2017 Fiona, Tim and I co-established a group, Transparency in Ecology and Evolution, and created a preprint server, EcoEvoRxiv, for ecology, evolution and conservation in 2019. Also, in 2020, along with our colleagues, Fiona and I established a multidisciplinary organisation, the Australian Reproducibility Network (AusRN), a sister network to the UK Reproducibility Network (UKRN), which has been improving research quality at many UK universities.

My lab’s former and current members have been involved a lot in starting SORTEE too, but I think Rose is the better person to tell this. Anyway, we still have a lot of work to do, and open science and transparency initiatives keep me very busy.

**Figure:**
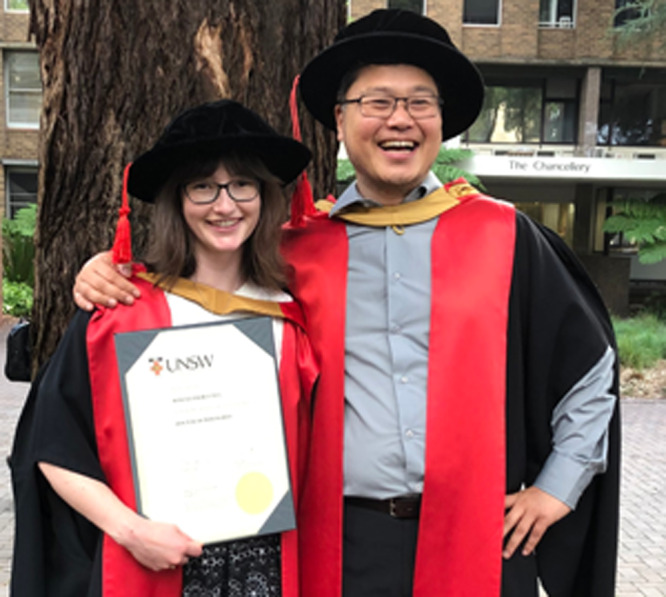
Dr Rose E. O’Dea & Prof Shinichi Nakagawa

**ROD:** Luckily, my first year of doing research in ecology and evolution was under the supervision of Michael Jennions and Megan Head, at the Australian National University in 2014. They were working on two projects about bias in published research: one on *p*-hacking^2^, and the other on experimental masking/blinding^3^. That meant that by the time I joined Shinichi’s lab at UNSW as a research assistant in 2015, the sharpest edges of my naive optimism about scientific publishing were blunted. I then learnt a lot more about problems with research, and remedies such as Registered Reports, at the workshop Shinichi mentioned, hosted by the Center for Open Science. But I started my PhD in 2016 with a different type of naivety: the belief that by working transparently and reproducibly, I’d do research that mattered. However, I committed premature hypothesis testing, and eventually came to understand that you can work transparently and reproducibly and still publish papers that fail to grow our collective understanding of how the world works.

Towards the end of my PhD I became more and more interested in conversations happening in other fields about the proliferation of pointless papers^4,5^, so I went to the first conference of the Association for Interdisciplinary Meta-research and Open Science (AIMOS). At that conference, Tim Parker was gauging interest from ecologists in forming an open science community, inspired by the Society for the Improvement of Psychological Science (SIPS). That meeting was the start of a year-long process to launch SORTEE, and I’ve subsequently poured most of my frustrations and optimism for science into helping SORTEE grow. I hope SORTEE will elevate researchers’ concerns about the behaviours that are rewarded by publishers, funders, and hirers and advocate for institutional changes that promote more meaningful research^6^. I don’t think more education and training is enough.

Many researchers in ecology and evolution may be unfamiliar with Registered Reports. Can you briefly explain what these are, and how they differ from preregistration?

A Registered Report is a peer-reviewed empirical publication where peer-review happens before the results of the study are known. Registered reports were first implemented in 2013 by Chris Chambers in the journal *Cortex*^7^, and their adoption by more and more journals is one of the most exciting changes to the traditional publication system we can remember. The exact requirements of a Registered Report can differ between journals, but all follow two basic stages.

In Stage 1, authors submit a study rationale and methods to a journal (e.g., imagine a normal manuscript, but without the results and discussion sections). Just like with traditional papers, if the editor thinks the study might be suitable for publication in the journal, they will send it out to peer-reviewers. Because the reviewing and revising process occurs before the study is conducted, the quality of the study might be improved following peer review. And unlike in traditional publications, reviewers must focus on the soundness of the study’s design and methods (which authors can control) instead of the neatness or sexiness of the results (which authors shouldn’t be able to control). Once the editor is satisfied that the planned study is suitable for the journal, the Registered Report receives an in-principle acceptance. In some journals, that ‘Stage 1’ manuscript is available online (helping other researchers find work-in-progress).

In Stage 2, the authors conduct the study as planned. In the simplest scenario, the authors follow their planned methods exactly, add the results and discussion sections to their unaltered Stage 1 manuscript, resubmit it for a quick round of peer-review, and the manuscript is accepted (regardless of how ambiguous or boring the results turn out to be). But of course, even the best-laid plans can go awry. Journals will differ in their response to deviations from the Stage 1 methods (e.g., authors might request an amendment to the Stage 1 report and/or include a ‘Deviations from Stage 1’ section in the final manuscript). Editors will decide whether the deviation is large enough to void the Stage 1 in-principle acceptance.

People often get Registered Reports confused with pre-registration (in hindsight, maybe they should have been named less similarly!). Pre-registration (or simply ‘registration’) is where the authors make a time-stamped, unalterable record of their study plans before they conduct the study, but it is not submitted to a journal for peer-review, and it does not receive in-principle acceptance. Pre-registration is, therefore, less costly than Registered Reports regarding authors’ time and effort, but it also doesn’t come with the same benefits (such as in-principle acceptance, and improvements to the study following review).

Why should researchers consider Registered Reports for their work?

Publication bias and selective reporting are huge problems across the sciences, including ecology and evolutionary biology. A recent meta-analysis estimated that over 40% of research projects were never published in ecology and evolution, which is pretty similar to an estimate of 50% from medicine^8^. When people preferentially publish exciting results or the studies that ‘work’, then our journals become filled with false positives. Some researchers waste years trying to build on non-replicable research. Others waste more years trying and failing to do something that was attempted by others without them knowing it because the failures weren’t published. Not only is this inefficient, there are also ethical issues to consider. Think of all the lives and feelings (including the study subjects) wrapped up in research.

In theory, registered reports can eliminate publication bias. For a registered report to be published, researchers must test the questions they planned (eliminating ‘HARKing’: Hypothesizing After the Results are Known^9^), conduct their analyses as planned (eliminating *p*-hacking), and present all results (eliminating selective reporting). In practice, the initial evidence on this is promising: the rate of null results rose from 10% in the traditional literature to 60% in registered reports^10^.

But aside from the community benefits of eliminating publication bias, there are also big personal benefits to the individual researcher in investing their time and energy on Registered Reports.

First, reduce uncertainty in whether, where, and when the study will be published. In the traditional publication system, it can be difficult to publish null or ‘messy’ results. Those results might stay in the file drawer, or the researchers might have to shop them around to multiple journals until they’re accepted (a tedious and drawn-out process). Moreover, authors, editors, or reviewers might be tempted to massage those results into a more compelling story. (SN has seen reviewers request changes to the introduction to better fit the results, i.e., HARKing). But for a Registered Report (that has received in-principle-acceptance), a journal has already agreed to publish those results, saving the authors time and worry. The added certainty might help the authors plan future work and make it easier to strategically time job or grant applications.

Second, the study design or methodology might be genuinely improved by reviewer comments. Indeed there is observational evidence that Registered Reports are rated as being of higher quality than traditional publications^11^. In the traditional mode of publication, it is usually too late to change methods when a reviewer critiques them. If you agree with their critique, what are you to do? Withdraw your manuscript, extend the limitations section, double down and try to explain the critique away? No great options there. In a Registered Report the suggested changes can be implemented. Those comments might save the authors from wasted time and effort on flawed data collection. Ultimately, conducting a higher-quality study should improve the authors’ professional reputation. And we could flip perspectives here: as well as being authors, we are also reviewers. Wouldn’t the experience of peer-reviewing feel more rewarding if it could improve the study that is ultimately published?

Finally, because Registered Reports force authors to clarify their ideas and methodology well ahead of time, they can be valuable training and mentoring tools for early career scientists and their advisors. However, this benefit can also be perceived as a cost, which we’ll talk about in the next section.

What do you think are the main challenges for Registered Reports in your field?

The most obvious barrier is time. The authors need to wait for in-principal acceptance before starting data collection. Many researchers work on short-term contracts, or under short funding cycles, and this waiting time goes against the advice to ‘hit the ground running’ (i.e. collect as much data as you can while you have funding, and write it up later). The waiting time is especially tricky for time-sensitive fieldwork in ecology and evolution, but there are possible ways around this. Depending on journal policies, there might also be time pressures on the deadline to submit Stage 2 after receiving in-principle acceptance (the Registered Reports we have been involved with all needed a deadline extension).

Like the above, research trainees often learn while doing and collect data before they know how to analyse it. How, then, does the student write a Registered Report? Two solutions come to mind. The student could lose a sense of ownership over their methods and have their adviser or a collaborator write the sections that they do not yet understand. Obviously, this is undesirable. Alternatively, the student could conduct a smaller pilot study to learn all the skills and gain all the knowledge they will need to write the Registered Report. The results from a pilot study can inform data simulations, to help pre-specify analysis plans and conduct power analyses. This option feels preferable but given the aforementioned time constraints on researchers (especially trainees), it will not always be practical.

Another challenge for Registered Reports is that many of their benefits depend upon peer-review working well. There are already entrenched problems with peer-review in the traditional publication system^12^. One of the problems—that editors struggle to find expert reviewers to cover the workload—might be slightly ameliorated with Registered Reports if they prevent authors from sending their manuscripts to several journals before acceptance. But there will be the additional problem that peer-reviewers are not always trained to engage deeply with the theoretical justification and study design of the manuscripts they assess Research fields are already filled with premature hypothesis testing^13^. Perhaps it is expecting too much of Registered Reports for peer-reviewers to curtail our culture of flimsy theory development.

The final challenges should be the easiest to fix: access and awareness. Few of the specialist ecology and evolution journals offer Registered Reports (those that do include *Ecology and Evolution, Ethology, Conservation Biology*, and *Ecological Solution and Evidence*). To try and address the shortage of participating journals, the 2021 SORTEE conference held a hackathon, led by a PhD student Patrice Pottier, to write to ~100 Editors-in-Chief of journals (building on an earlier campaign by Hannah Fraser). We had limited success, and there were frequent misunderstandings about Registered Reports among editors (some of which we’ll talk about in the next section). The good news was that *Nature Communications* was already planning to accept Registered Reports in all fields, including ecology and evolution (before, it was limited to cognitive neurosciences, psychology, social science and epidemiology articles).

Are there any specific areas that you think are particularly suitable for Registered Reports, or any advice you’d give to authors?

Given the challenges outlined above, the most suitable studies to write as a Registered Report are those for which: (1) you have a strong study rationale; (2) you have well-developed methods; and (3) you can afford to wait for in-principle acceptance before starting data collection.

Note that these criteria extend beyond experimental or hypothesis-testing research. We think that observational research can be just as well suited to Registered Reports.

We also want to emphasise that Registered Reports do not prevent serendipitous discoveries. One of the main purposes of Registered Reports, and registration in general, is to distinguish predictions (one’s initial plan) from postdictions (unplanned discoveries). The post-hoc discoveries can either be reported in the Registered Report (as a deviation from Stage 1 methods), or they may form the basis for a separate paper.

Finally, a word of encouragement. At the SORTEE conference this year, a piece of feedback was that it can be stressful to hear about new open science initiatives, as there are so many of them, and it’s hard to know where to start. But the first step is being curious, and then it’s one step at a time after that. (Pre-)registration is a good place to start (see here), as this process can still help you avoid questionable research practices such as HARKing and *p*-hacking. But If you do decide to submit a Registered Report, then you will be among the early adopters of a revolution in scientific publishing that will hopefully last long into the future.

*This interview was conducted by Walter Andriuzzi.*
